# Complementary and Alternative Therapy of Rare Inflammatory/Autoimmune Diseases

**DOI:** 10.1155/2018/2140521

**Published:** 2018-03-07

**Authors:** Young-Su Yi

**Affiliations:** Department of Pharmaceutical Engineering, Cheongju University, Cheongju 28503, Republic of Korea

Inflammation is a series of biological processes to protect our body from harmful stimuli, including pathogens, damaged cells, and a variety of irritants. Although inflammation is the protective response, prolonged inflammation, known as chronic inflammation triggered and sustained by unknown reasons, leads to a progressive recruitment and activation of the inflammatory immune cells present at the inflammatory sites and is characterized by “stuck” in the active sites and simultaneous destruction of the tissues, finally resulting in inflammatory/autoimmune diseases.

Inflammatory/autoimmune diseases have been intensively studied for a long time, and recently, some inflammatory/autoimmune diseases which are very common in worldwide, including rheumatoid arthritis and psoriasis, have achieved an extraordinary progress in scientific understanding as well as drug development for a therapeutic purpose. However, a large subset of inflammatory/autoimmune diseases that are less common but more serious have not been focused on and poorly explored. These are categorized as “rare inflammatory/autoimmune diseases,” indicating that they affect small number of patients. There are over 150 rare inflammatory/autoimmune diseases, many of which are incurable and terminal.

In this special issue, we invited investigators to contribute original research articles and review articles that will help understand the basic mechanisms as well as the development of new and promising complementary and alternative strategies to diagnose and treat the rare inflammatory/autoimmune diseases. Seven studies were published regarding the complementary and alternative therapy of rare inflammatory/autoimmune diseases.

The research article by Q. Xu et al. demonstrated the therapeutic effects and mechanisms of Qi-Wu Rheumatism Granule (QWRG), a Chinese herbal compound on adjuvant-induced RA in rats, and suggested that the antiarthritic properties of QWRG may be due to immunodepression and downregulation of inflammatory cytokines, which may be a potential candidate for the treatment of rheumatoid arthritis.

The research article by X. Deng et al. demonstrated that the effect and possible mechanism of icariin, a prenylated flavonol glycoside derived from the Chinese herb,* Epimedium sagittatum*, on the IL-1*β* pretreated human nucleus pulposus cells and suggested that icariin might be a protective traditional Chinese medicine, which prevent inflammation-induced degeneration of intervertebral discs partly through the PI3K/AKT pathway.

The research article by C. Lv et al. demonstrated the antibacterial function of Liu-He-Dan (LHD), a traditional Chinese medicine used to treat infective wounds by clinical and biomolecular researches, and suggested that LHD could specifically suppress the expression of IL-1*β* and upregulate the expression of basic fibroblast growth factor (bFGF) in the wounds, promoting the healing of infective wounds by decreasing the release of inflammatory factors from the infective wounds.

The research article by B. Ji et al. demonstrated the effects and underlying mechanism of Jinkui Shenqi pills (JKSQP), a popular formula with kidney warming and yang enhancing effects in a rat model of asthma with kidney-yang deficiency (KYD), and provided a basis for the development of JKSQP as a novel therapeutic agent to treat asthma.

The research article by J. Li et al. demonstrated the pharmacological effect of Sheng-jiang powder (SJP), a classic representative Chinese medicine formula to treat “ascending and descending dysfunction of spleens” that has been considered the primary cause of obesity on the pathogenesis of obesity and obesity-mediated multiorgan injuries in high fat diet-induced obese rats and suggested that SJP ameliorates inflammatory response in tissues of obese rats and mitigates obesity-induced multiple organ injuries.

Finally, the review article by X.-Q. Wang et al. described the Chinese medical theories relating to the pathogenesis of refractory nephrotic syndrome (RNS) and discussed the strategies and treatment options using Chinese herbal medicines. This review provided an insight and great potential of traditional Chinese medicines for the prevention and treatment of RNS.

We hope that readers will be interested in rare inflammatory/autoimmune diseases and that this special issue could attract the interest of scientific community in order to improve further investigations leading to the discovery of novel biomarkers in the field of rare inflammatory/autoimmune diseases.

*Young-Su Yi*

